# Mate fidelity in a polygamous shorebird, the snowy plover (*Charadrius nivosus*)

**DOI:** 10.1002/ece3.5591

**Published:** 2019-09-04

**Authors:** Naerhulan Halimubieke, José O. Valdebenito, Philippa Harding, Medardo Cruz‐López, Martín Alejandro Serrano‐Meneses, Richard James, Krisztina Kupán, Tamás Székely

**Affiliations:** ^1^ Department of Biology and Biochemistry, Milner Centre for Evolution University of Bath Bath UK; ^2^ Posgrado en Ciencias del Mar y Limnología Universidad Nacional Autónoma de México, Ciudad Universitaria Cd. México Mexico; ^3^ Departamento de Ciencias Químico‐Biológicas Universidad de las Américas Puebla San Andrés Cholula Puebla Mexico; ^4^ Department of Physics and Centre for Networks and Collective Behaviour University of Bath Bath UK; ^5^ Max Planck Institute for Ornithology, Behaviour Genetics and Evolutionary Ecology Research Group Seewiesen Germany; ^6^ Department of Evolutionary Zoology and Human Biology University of Debrecen Debrecen Hungary

**Keywords:** breeding dispersal, *Charadrius nivosus*, divorce, mate fidelity, nesting success, polygamous

## Abstract

Social monogamy has evolved multiple times and is particularly common in birds. However, it is not well understood why some species live in long‐lasting monogamous partnerships while others change mates between breeding attempts. Here, we investigate mate fidelity in a sequential polygamous shorebird, the snowy plover (*Charadrius nivosus*), a species in which both males and females may have several breeding attempts within a breeding season with the same or different mates. Using 6 years of data from a well‐monitored population in Bahía de Ceuta, Mexico, we investigated predictors and fitness implications of mate fidelity both within and between years. We show that in order to maximize reproductive success within a season, individuals divorce after successful nesting and re‐mate with the same partner after nest failure. Therefore, divorced plovers, counterintuitively, achieve higher reproductive success than individuals that retain their mate. We also show that different mating decisions between sexes predict different breeding dispersal patterns. Taken together, our findings imply that divorce is an adaptive strategy to improve reproductive success in a stochastic environment. Understanding mate fidelity is important for the evolution of monogamy and polygamy, and these mating behaviors have implications for reproductive success and population productivity.

## Graphical abstract

In sequential polygamous snowy plover, with limited breeding time, individuals divorce after successful nesting and re‐mating to the same partner after nest failure to maximize reproductive success within a season.

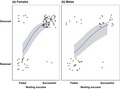

## 1 INTRODUCTION

The decision of retaining a mate for several breeding events or divorcing is a key element of reproductive decisions in several species, as it can affect reproductive success and subsequent survival of the parents (Culina, Radersma, & Sheldon, 2014; Neff & Pitcher, 2005; Székely, Thomas, & Cuthill, 2006; Székely, Weissing, & Komdeur, 2014). Social monogamy, defined as a system where an adult has only one social partner of the opposite sex at a given time or throughout a time period, is commonly observed in birds, but also occurs in invertebrates, fish, amphibians, reptiles, and mammals (Lukas & Clutton‐Brock, 2013; Møller, 2003). Social monogamy partnerships are highly variable in terms of duration. Some species show long‐term mate fidelity or even life‐time mate fidelity until one partner dies (Black, 2001; Reichard & Boesch, 2003). Other species, however, exhibit short‐term mate fidelity, in which an individual terminates the relationship at the end of one breeding attempt and initiate another breeding with a new mate while the old partner is still alive (termed, sequential polygamy). Why do males and females adopt short‐term mate fidelity, while others pair for life?

Several hypotheses have been put forward emphasizing the impact of either breeding time‐constraints (or breeding success) on mate fidelity or divorce in socially monogamous species. On the one hand, retaining a mate reduces the time and energy costs of searching for a new mate therefore facilitate a fast re‐mating (“fast‐track hypothesis,” Adkins‐Regan & Tomaszycki, 2007; Perfito, Zann, Bentley, & Hau, 2007). Retaining a mate also enhance breeding performance thereby improving reproductive success (“mate familarity hypothesis,” Ens, Choudhury, & Black, 1996; Gabriel, Black, & Foster, 2013; Sánchez‐Macouzet, Rodríguez, & Drummond, 2014). In addition, successful breeding may also facilitate retaining the mate for future breeding (Black, 2001; Flodin & Blomqvist, 2012). On the other hand, changing a mate may be beneficial in long‐lived species, individuals divorce their partner to mate with good quality partners in order to improve breeding success (“incompatibility hypothesis,” Coulson, 1966; see also Kempenaers, Adriaensen, & Dhondt, 1998). In species with short life span (or short breeding season), individuals improve reproductive success by mating with multiple mates to make the most out of limited time (“extra‐pair mating hypothesis,” Arnqvist & Nilsson, 2000; Birkhead & Møller, 1992).

Mating decisions may be related to breeding dispersal—the latter defined here as the movement of an adult from one breeding location to another within or between years (Clobert, Danchin, Dhondt, & Nichols, 2001; Greenwood, 1980). On the one hand, breeding dispersal may differ between the sexes in response to sex differences in mating strategies since the more polygamous sex is expected to disperse farther to find new mating partners (D'Urban Jackson et al., 2017; Greenwood, 1980; Székely, 2019; Trochet et al., 2016). On the other hand, mate fidelity can be viewed as a by‐product of site fidelity in some species (Bried, Pontier, & Jouventin, 2003; Morse & Kress, 1984), whereas changing the nest site would lead to mate change in some other species (Pietz & Parmelee, 1994; Thibault, 1994).

A further factor that may influence mate fidelity is re‐mating opportunity. In species or populations with a biased adult sex ratio, divorce is commonly initiated by the rare sex since the rare sex has higher mate availability than the common sex (Liker, Freckleton, & Székely, 2014; Parra, Beltrán, Zefania, Dos Remedios, & Székely, 2014). For example, experimental studies of species with biased adult sex ratio showed that by experimentally creating unmated males and females, re‐mating times were shorter for rare sex than for common sex (Parra et al., 2014; Székely, Cuthill, & Kis, 1999).

Nevertheless, studies of mate fidelity tended to focus on monogamous species across breeding years, yielding different adaptive implications of mate fidelity (Bried et al., 2003; Dubois & Cézilly, 2002). Monogamous systems are generally characterized by high level of breeding philopatry (Moore & Ali, 1984; Saalfeld & Lanctot, 2015) and/or bi‐parental care of the young (Eberhart‐Phillips et al., 2018), features that tend to promote mate fidelity. However, the causes and fitness implications of mate fidelity in sequential polygamous species that exhibit variable duration of pair bonds (e.g., within a breeding year), different levels of philopatry, or breeding dispersal are still poorly understood.

Here, we investigate potential predictors and fitness implications of mate fidelity in a sequential polygamous shorebird, the snowy plover (*Charadrius nivosus*), a ground‐nesting, near threatened shorebird distributed on sparsely vegetated coasts and alkaline lakeshores across the temperate and tropical regions of the Americas. They typically lay a 3‐egg clutch with both parents providing care during the incubation stage, chicks are precocial and nidifugous, which only require uniparental care (usually males) during brood rearing (del Hoyo, Elliott, Sargatal, Christie, & Juana, 2018). This species is an ideal model for investigating mate fidelity: they have a flexible mating system, and both males and females may have several mates sequentially in a single breeding season up to four breeding attempts (Page, Stenzel, Warriner, Warriner, & Paton, 2009). It is typically females that mate with more partners than males do, since females tend to desert their broods soon after hatching, and leave the males to look after the young until independence (Carmona‐Isunza et al., 2017; Warriner, Warriner, Page, & Stenzel, 1986). Female desertion has been linked to male‐biased adult sex ratio (ASR): 0.53 (proportion of males in the adult population) was estimated by Stenzel et al. (2011) based on adult survival, whereas more recent estimate that took into account hatchling sex ratios, chick survival and adult survival estimated a strongly male‐biased ASR (0.638, Eberhart‐Phillips et al., 2018). Snowy plovers may still retain their mate between clutches within or between years. Furthermore, a recent paternity analyses showed low rates (<5%) of extra‐pair paternity in the snowy plover so that social pairs are a good proxy for genetic relationships and thus reflect Darwinian fitness (Maher et al., 2017).

Using snowy plovers as a model organism, here we investigate whether mate fidelity (or divorce) is an adaptive strategy that maximizes reproductive success in a species with limited breeding period (Choudhury, 1995; Plaschke, Bulla, Cruz‐López, Gómez del Ángel, & Küpper, 2019). We focus on three main aspects of mate fidelity. First, we investigate patterns of mate fidelity both within and between years in both males and females. Second, we explore if previous nesting success predict mate retention (or divorce) by males and females both within and between years. Finally, we investigate mate fidelity in relation to breeding dispersal and re‐mating time: (a) whether breeding dispersal is related to mate fidelity both within and between years; (b) whether the re‐mating time may differ between divorced and retained mates within years.

## 2 METHODS

### 2.1 Study site and field methods

The present study was conducted at Bahía de Ceuta, Sinaloa, Mexico (23°54′N, 106°57′W). In this population, snowy plovers nest on extensive saline ponds and saltpans (approximately 150 hectares; Carmona‐Isunza, Küpper, Serrano‐Meneses, & Székely, 2015). The breeding season generally occurs from mid‐April to mid‐July, with 30–100 breeding pairs every year. Breeding data were collected from 2006 to 2011 (*n* = 625 nests). Data collection in the field followed the methods of Székely, Kosztolányi, and Küpper (2008). Briefly, we searched for nests using a mobile hide intensively within the study site, we recorded the nest location with handheld GPS, and the egg‐laying date was estimated based on the floatation stage of each egg in a transparent jar with clean water. Breeding pairs were captured with a walk‐in funnel trap placed over the nest, and they were banded with a unique combination of three color rings and an alpha‐numeric metal ring. Nests were monitored every 2–5 days until 20 days of incubation and then were checked every day until hatching to obtain nesting success data. Broods were searched intensively daily to determine the date of brood desertion. Re‐sightings of previously color banded plovers were also recorded.

### 2.2 Data collection

#### 2.2.1 Quantification of mate fidelity

Snowy plovers that were monitored in this study were actively choosing to retain or to divorce their mates. The mating decision of each individual was recorded as either mate retention or divorce in regard to their previous breeding attempt. We evaluated mating decisions separately for banded males and females in the population, since the decisions may influence one another and as such may not be independent. Individuals were included in the analyses if they satisfied the following conditions: (a) we knew the identity of their mate(s), (b) they were observed in at least two reproductive attempts that were either within or between years, and (c) if there is a mate change, only those who change their mates while the previous mate is known to be alive are included. In total, 149 breeding events (Table 1A, 75 divorces in females, 26 divorces in males, and 24retentions in each sex) fitted the criterion for the within‐year mate fidelity analysis from 2006 to 2011. For plovers with more than two nests within a year, only the data from the first two nests were included in the within‐year mate fidelity analysis due to the small number of individuals with three or more nests: during the study period, there were only seven females and two males in total that had three breeding attempts.

**Table 1:** Mate fidelity in snowy plover. (A) Number of males and females divorced or retained a mate within years, *n* = 149 breeding events. (B) Number of males and females divorced or retained a mate between breeding years (late–early mate fidelity, *n* = 102 breeding events; early–early mate fidelity, *n* = 116 breeding events; 2006–2011)

(A) Within years							
Year	2006	2007	2008	2009	2010	2011	Total
Number of divorces in females	11	21	10	14	12	7	75
Number of retentions in females	6	8	3	2	3	2	24
Number of divorces in males	5	8	3	3	4	3	26
Number of retentions in males	6	8	3	2	3	2	24

(B) Between years						
Year	2006–2007	2007–2008	2008–2009	2009–2010	2010–2011	Total
late–early mate fidelity						
Number of divorces in females	12	6	7	12	5	42
Number of retentions in females	4	1	1	3	2	11
Number of divorces in males	8	8	11	7	4	38
Number of retentions in males	4	1	1	3	2	11
early–early mate fidelity						
Number of divorces in females	13	4	4	7	7	35
Number of retentions in females	1	4	2	6	3	16
Number of divorces in males	17	7	8	7	10	49
Number of retentions in males	1	4	2	6	3	16

Note

See Section 2 for details.

**Figure 1:**
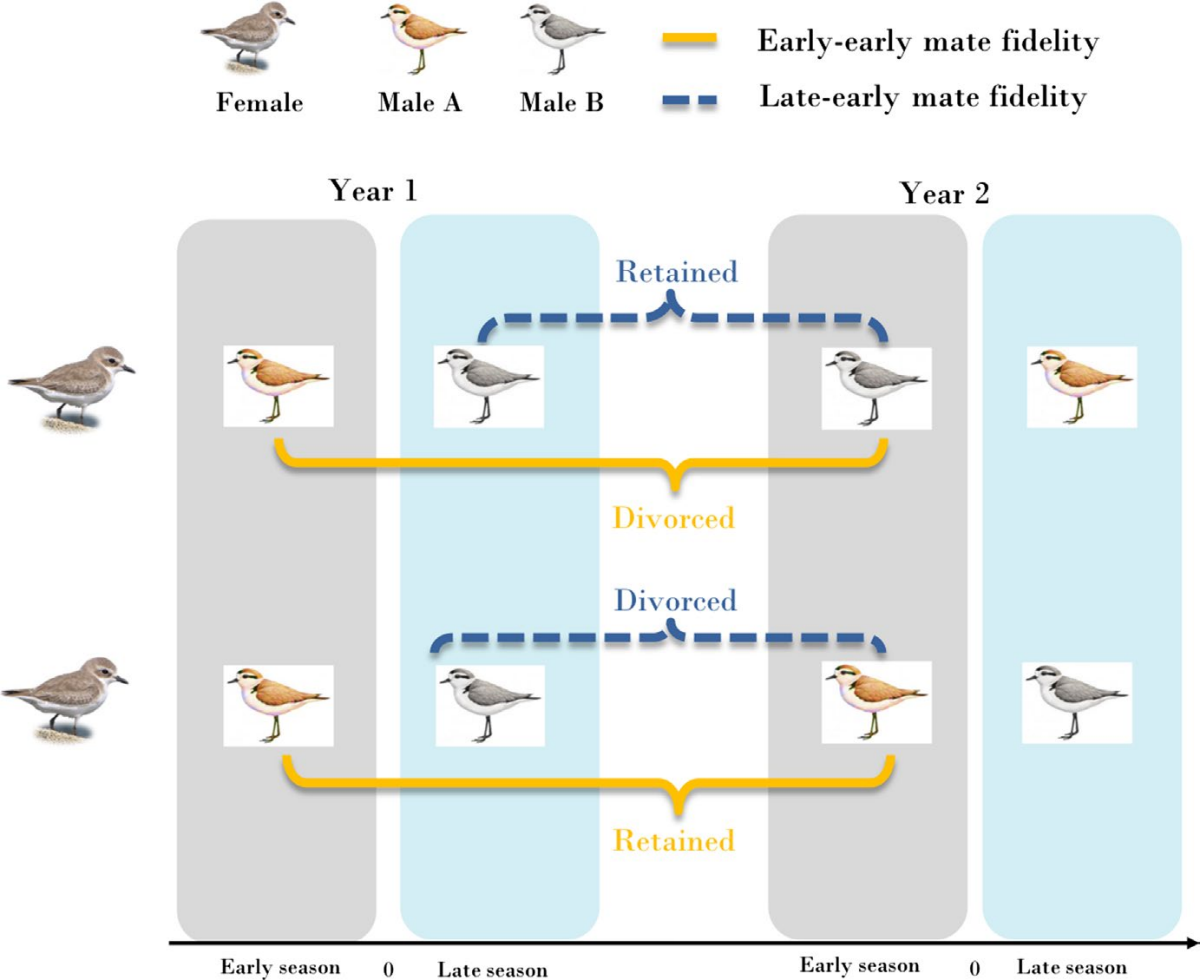
Schematic illustration of two estimates of between‐year mate fidelity in snowy plovers: Early‐late and early–early mate fidelities

For individuals with one or multiple nests in each of the two consecutive years, we evaluate between‐year mate fidelity in two different ways (see Figure 1). First, when an individual's mate during late season (see “relative egg‐laying date” below, late season is when the relative egg‐laying date is >0) in year 1, had the same as the mate in early season (when the relative egg‐laying date is <0) in year 2, it was classified as mate retention, or otherwise divorce (hereinafter late–early mate fidelity). In total, 102 breeding events (Table 1B, 42 divorces in females, 38 divorces in males and 11 retentions in each sex) fitted the criteria for the late–early mate fidelity. Second, if a plover mated to the same individual in the early seasons of both year 1 and year 2, this was classified as retention, or divorce otherwise (hereinafter early–early mate fidelity). In total, 116 breeding events (Table 1B, 35 divorces in females, 49 divorces in males and 16 retentions in each sex) fitted the criteria for the early–early mate fidelity. All individuals were classified into three groups as divorced males, divorced females, and retained pairs (see Sandercock, Lank, Lanctot, Kempenaers, & Cooke, 2000).

#### 2.2.2 Nesting success and reproductive success

Nesting success was quantified based on the fate of the first nest of each individual that were included in our study. The fate of nest was recorded as either successful (at least one chick hatched) or failed (no chicks hatched due to predation, destruction, abandonment, eggs disappeared <15 days after estimated laying date, eggs did not hatch, or the nest was flooded). We quantified reproductive success as the cumulative number of hatchlings each individual produced in all breeding attempts either within or between years.

#### 2.2.3 Relative egg‐laying date

The egg‐laying date was used to quantify breeding phenology. We controlled for breeding phenological differences between years by converting egg‐laying dates into Julian dates (“lubridate” package in R, Grolemund & Wickham, 2011), and calculating the relative egg‐laying date using the z‐transformation (mean = 0, *SD* = 1).

#### 2.2.4 Breeding dispersal

Within‐year breeding dispersal was defined as the straight‐line distance (in meters) between an individual's successive nests within a year. For between‐year breeding dispersal, we measured the straight‐line distance between (a) the last nest in year 1 and the first nest in year 2, and (b) the first nests of two consecutive years.

#### 2.2.5 Re‐mating time

Re‐mating time is defined as the number of days that an individual spent on establishing a new clutch after terminating care of the previous brood. Broods were searched in the breeding area daily. If a parent was missing during two consecutive sightings or seen paired to another plover, it was considered to have deserted the brood. We estimated the date of brood desertion for a parent as the mid‐point between the time when the individual was last seen with his/her brood and first seen without the brood. We estimated second nest egg‐laying date based on the floating stage of the eggs (see above). We only estimated the re‐mating times within years.

### 2.3 Statistical analyses

#### 2.3.1 Comparison of male and female mate fidelity

We analyzed mating decision as either mate retention or divorce of a plover from an individual its previous breeding attempt. We calculated the number of mate retentions and number of divorces in males and females within the population for both within and between years. We used the two‐proportion *z* test (Yau, 2013) to compare the proportion of divorced females relative to the female population to the proportion of divorced males relative to the male population both within and between years.

#### 2.3.2 The relationship between mate fidelity and nesting success

We constructed separate models for males and females to investigate whether mate fidelity is related to nesting success within and between years. Here, separation of the sexes was necessary since nesting success is nonindependent variable within a pair; therefore, individuals of a pair provide the same data points. In the latter analyses, mate fidelity of an individual was the dependent variable, and nesting success was used as explanatory variable. To analyse the females, we used generalized linear mixed models (GLMM) with binomial error and included Individual ID and Year as random effect variables to account for the repeated identities of females among years. For males, we used generalized linear model (GLM) with binomial error.

#### 2.3.3 Reproductive success and mate fidelity

To investigate whether mate fidelity relates to reproductive success (estimated as the total number of hatchlings from both clutches), we compared divorced males, divorced females, retained pairs using Kruskal–Wallis tests followed by post hoc pairwise comparisons (Dunn test) to test group differences within and between years.

#### 2.3.4 Breeding dispersal and mate fidelity

Models were built to investigate the relationship between breeding dispersal and mate fidelity groups within and between years. Log‐transformed (ln) breeding dispersal was the dependent variable, and mate fidelity groups (divorced males, divorced females, and retained pairs) were the explanatory variable. Linear mixed‐effects model (LMM) via REML was fitted and maintained Individual ID and Year as random effect variables. Then, the estimated marginal means (emmeans from package “emmeans” in R) were calculated for each group, post hoc pairwise comparisons adjusted by Tukey were applied to test group differences.

#### 2.3.5 Re‐mating time and mate fidelity

To investigate whether re‐mating time differs between mate fidelity groups (divorced males, divorced females, and retained pairs), we used Kruskal–Wallis tests followed by post hoc pairwise comparisons (Dunn test) to test group differences within and between years. All statistical analyses were performed using R version 3.5.1 (R Core Team, 2018).

## 3 RESULTS

### 3.1 Mate fidelity between sexes

Within breeding years, males showed higher mate fidelity than females using 149 breeding events (Table 1A, 75 divorces in females, 26 divorces in males, and 24 retentions in each sex) from 2006 to 2011, two‐proportion *z* test, *p* = .002, *n* = 6 years). The different numbers of female and male breeding attempts are due to the fact that more females than males had multiple breeding attempts. Between breeding years, however, we did not find a difference in mate fidelity of males versus females (Table 1B, two‐proportion *z* test; late–early mate fidelity: *p* = 1.00, *n* = 5 years; early–early mate fidelity: *p* = .55, *n* = 5 years).

### 3.2 Mate fidelity in relation to nesting success and reproductive success

**Figure 2:**
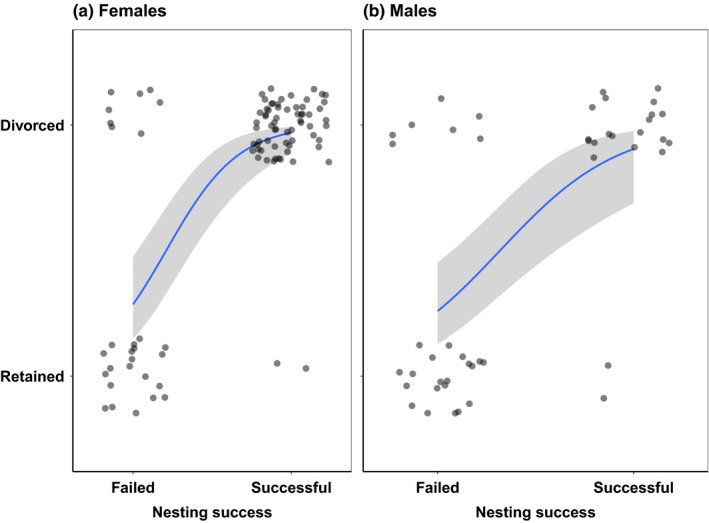
Mate fidelity in relation to nesting success in (a) female and (b) male snowy plovers within a year (see Table 2 for statistics). Logistic linear regression lines (blue) with standard error (gray)

Within breeding years, mate fidelity was related to nesting success since divorce was more likely when the nest hatched successfully, whereas mate retention was more likely if the nest failed (Table 2, females: GLMM, *p* < .001, male: GLM, *p* < .001; Figure 2). Between breeding years, however, mate fidelity was not related to nesting success. The latter result was consistent between the late–early mate fidelity and early–early mate fidelity (Table 2).

**Table 2:** Mate fidelity in relation to nesting success within and between breeding years in snowy plover

Response variable	Model used	Explanatory variable	Estimate	*SE*	*z* value	*p* value
Within years						
Female						
Mate fidelity	*Binomial* (GLMM)	Intercept	−0.92	0.42	−2.19	**.03**
		Nesting success	4.38	0.83	5.27	**<.001**
Male						
Mate fidelity	*Binomial* (GLMM)	Intercept	−15.92	5.16	−3.09	**.002**
		Nesting success	29.17	7.59	3.84	**<.001**
Between years: late–early mate fidelity						
Female						
Mate fidelity	*Binomial* (GLMM)	Intercept	1.39	0.79	1.75	.08
		Nesting success	−0.06	0.87	−0.07	.95
Male						
Mate fidelity	*Binomial* (GLM)	Intercept	1.50	0.78	1.92	.05
		Nesting success	−0.33	0.87	−0.38	.70
Between years: early–early mate fidelity						
Female						
Mate fidelity	*Binomial* (GLMM)	Intercept	1.47	1.19	1.23	.22
		Nesting success	−0.75	1.25	−0.60	.55
Male						
Mate fidelity	*Binomial* (GLM)	Intercept	2.30	1.05	2.20	**.03**
		Nesting success	−1.37	1.10	−1.26	.21

Note

Generalized linear mixed models (GLMM) with *binomial* error family and including “Individual ID” and “Year” as random effect variables to account for the repeated identities of female individuals among years. For males, generalized linear model (GLM) with *binomial* error family was used.

Abbreviation: *SE*, standard error.

Statistically significant results are presented in bold.

**Figure 3:**
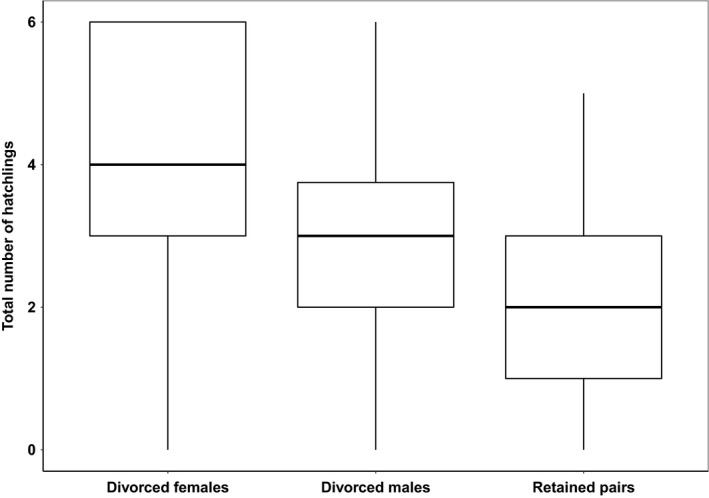
Reproductive success in relation to divorce or mate fidelity in snowy plovers (see Table 3 for statistics). Medians, upper, and lower quartiles, as well as extreme values are shown

Divorced plovers (both males and females) produced significantly more hatchlings within breeding years than those retained their mate. Reproductive success was not different between divorced males and divorced females (Table 3, Kruskal–Wallis tests, *p* < .001, followed by post hoc pairwise Dunn test; divorced females—retained pairs: *p* adjusted < .001, divorced males—retained pairs: *p* adjusted = .05, divorced females—divorced males: *p* adjusted = .07; Figure 3). Between breeding years, however, reproductive success was not different between divorced and retained individuals neither in the late–early nor in the early–early comparisons (Kruskal–Wallis tests; late–early mate fidelity: χ^2^ = 0.20, *df* = 2, *p* = .90; early–early mate fidelity: χ^2^ = 4.21, *df* = 2, *p* = .12).

**Table 3:** Comparison of reproductive success between mate fidelity groups (divorced males, divorced females, and retained pairs) within breeding years (Kruskal–Wallis tests, *p* < .001, followed by post hoc pairwise Dunn test)

Groups	*Z*	*p* unadjusted	*p* adjusted
Within years			
Divorced females—divorced males	1.97	<.001	.07
Divorced females—retained pairs	4.08	<.001	**<.001**
Divorced males—retained pairs	1.92	<.001	**.05**

Statistically significant results are presented in bold.

### 3.3 Mate fidelity in relation to breeding dispersal and re‐mating time

**Figure 4:**
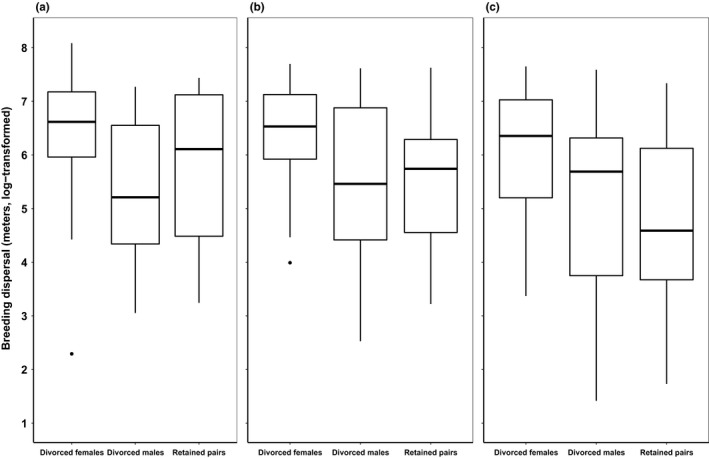
Breeding dispersal (a) within year, and between year (b, late–early) and (c, early–early) in snowy plover (see Section 2 for explanations and Table 4 for statistics). Breeding dispersal was estimated in meters and log‐transformed (ln). Medians, upper, and lower quartiles, as well as extreme values are shown

Divorced females bred further away than divorced males both within and between years (Figure 4, Table 4). Divorced males, however, did not breed further away than retained pairs (Table 4).

**Table 4:** (A) Breeding dispersal in relation to mate fidelity groups (divorced males, divorced females, and retained pairs) within and between breeding years. (B) Comparison of breeding dispersal between mate fidelity groups (divorced males, divorced females, and retained pairs) within and between breeding years

(A)					
Response variable	Model used	Explanatory variable	Estimate	*SE*	*t* value
Within years					
Breeding dispersal	LMM	Intercept	6.46	0.16	38.85
		Divorced males	−0.95	0.26	−3.63
		Retained pairs	−0.67	0.26	−2.58
Between years: late–early					
Breeding dispersal	LMM	Intercept	6.41	0.21	30.25
		Divorced males	−1.01	0.30	−3.37
		Retained pairs	−0.70	0.39	−1.77
Between years: early–early					
Breeding dispersal	LMM	Intercept	5.87	0.29	20.48
		Divorced males	−0.95	0.38	−2.53
		Retained pairs	−0.73	0.33	−2.23

(B)					
Groups	Estimate	*SE*	*df*	*t* ratio	*p* value
Within years					
Divorced females—divorced males	0.95	0.26	111	3.60	**.001**
Divorced females—retained pairs	0.67	0.26	112	2.57	**.03**
Divorced males—retained pairs	−0.28	0.26	68	−1.05	.55
Between years: late–early					
Divorced females—divorced males	1.01	0.31	72	3.28	**.005**
Divorced females—retained pairs	0.70	0.41	72	1.70	.21
Divorced males—retained pairs	−0.32	0.44	85	−0.71	.76
Between years: early–early					
Divorced females—divorced males	0.95	0.38	70	2.51	**.04**
Divorced females—retained pairs	0.73	0.35	56	2.09	.10
Divorced males—retained pairs	−0.22	0.44	88	−0.50	.87

Note

The linear mixed‐effects model (LMM) via REML was fitted and maintained “Individual ID” and “Year” as random effect variables.

Abbreviation: *SE*, standard error.

Statistically significant results are presented in bold.

Finally, re‐mating times were not different between divorced males, divorced females and retained pairs (Kruskal–Wallis test, χ^2^ = 2.00, *df* = 2, *p* = .37).

## 4 DISCUSSION

Previous analyses of mate fidelity were typically concerned with either within‐year or between‐year mate fidelity and focus largely on monogamous systems (sometimes termed mate desertion, mate abandonment or mate change; Black, 2001; Bried et al., 2003; Flodin & Blomqvist, 2012). Here, we take an integrative approach and investigate mate fidelity both within and between breeding years. Using a sequential polygamous shorebird, the snowy plover, we identified factors that predict mate fidelity and its spatial‐temporal manifestation in a system, in which males and females differ in their breeding strategies and reproductive efforts.

Our analyses revealed three major results. First, males exhibit higher within‐year mate fidelity than females. This is consistent with the previous studies of snowy plover since females tend to desert the brood whereas males are usually the ones that rear the young (Carmona‐Isunza et al., 2015; Warriner et al., 1986). We suggest that male‐biased adult sex ratio entices female parents more than male parents to desert their brood and breed again (Eberhart‐Phillips et al., 2017; Stenzel et al., 2011); thereby resulting in different re‐mating opportunities and mate fidelities between males and females. The latter results are consistent with experimental and empirical studies that show altered adult sex ratios influences mating decisions (Karlsson, Eroukhmanoff, & Svensson, 2010; Liker, Freckleton, & Székely, 2013; Liker et al., 2014; Silva, Vieira, Almada, & Monteiro, 2010).

However, between years both male and female snowy plovers demonstrated low mate fidelity. We note however that our mate fidelity (and consequently, our divorce decision as well) was based on local returning rates: if paired birds may breed outside the study area and/or some of the survived adults may not return to breed to Ceuta, these survival estimates can be biased. The annual return rate to Ceuta are 41.5% for males (*n* = 378 individuals) and 35.4% for females (*n* = 339 individuals, 2006–2011). Therefore, further investigation is required to estimate more precisely the return rates using more comprehensive spatial coverage by visiting additional breeding sites near Ceuta and/or using GPS tags to monitor the movements of adults within and between years.

Second, divorce was more likely after a nest hatched than after it failed since failed breeders typically re‐nested with the same partner. Therefore, divorced plovers, counterintuitively, reared more offspring than faithful individuals. This finding is not consistent with studies of long‐lived bird species where low breeding success may trigger divorce (“incompatibility hypothesis,” Black, 2001; Coulson, 1966; Jouventin & Bried, 2001). We propose that by abandoning the brood and divorcing, individuals try to maximize their reproductive success by producing as many clutches over the season as possible. Divorce may be facilitated by two aspects of natural history: first, nest and chick mortality in this population tend to be high and stochastic, and thus, parents may need several trials to produce at least some fledglings (Cruz‐López, Eberhard‐Phillips, et al., 2017). Second, the chicks are precocial, and thus, they only require modest care: brooding and protection, but not feeding (Székely & Cuthill & Kis, 1999). The well‐developed hatchling then gives the opportunity for one parent to terminate care and start breeding with a new partner (Houston, Székely, & McNamara, 2013; McNamara, Forslund, & Lang, 1999; Székely, Webb, Houston, & McNamara, 1996). Mate retention was, however, more likely after nest failure, in which case the parental duties of both parents terminated at the same time; therefore, the fastest way to breed again was to retain the previous partner (“fast‐track hypothesis”; Perfito et al., 2007; Zann, 1994; reviewed by Fowler, 1995; also see Adkins‐Regan & Tomaszycki, 2007).

However, breeding success in previous years may have little impact on the re‐mating decision of snowy plovers. We presume that the breeding time constraint facilitates early breeding with available mates instead of waiting for the former partner, especially since early breeding is associated with higher nest survival (Plaschke et al., 2019; van de Pol, Heg, Bruinzeel, Kuijper, & Verhulst, 2006; Székely et al., 1999). Since snowy plovers only have about 2 years of breeding life (average breeding life of males: 2.3 ± 1.6 years; females: 1.9 ± 1.2 years; Colwell, Pearson, Eberhart‐Phillips, & Dinsmore, 2013), they may not discriminate against previous mates even if they were failed breeders Furthermore, returning to the breeding ground may be stochastic and this can also produce decoupling between nesting success and mate fidelity (Bried, Frédéric, & Jouventin, 1999; Gilsenan, Valcu, & Kempenaers, 2017; Handel & Gill, 2000).

Third, we found that females tend to disperse farther than males after divorce both within and between breeding years. This follows the general pattern of female‐biased breeding dispersal observed in most bird species including shorebirds (Clarke, Saether, & Roskaft, 1997; Greenwood & Harvey, 1982; Liu & Zhang, 2008; Sandercock et al., 2000). However, in polyandrous birds like snowy plovers there is an additional reason: finding new mate while their previous mate is taking care of the chicks (D'Urban Jackson et al., 2017). For males, returning to previous breeding site—that is often thought as a high‐quality site providing good brood‐rearing opportunities (Sandercock et al., 2000)—is a factor that reduces their aptitude moving large distance between nests. Mate fidelity is often related to the degree of site fidelity (Cézilly, Dubois, & Pagel, 2000; Cézilly & Johnson, 1995), and while it would be tempting to argue that higher mate fidelity lead to higher site fidelity in males, or vice versa high divorce rate by females lead to more extensive breeding dispersal, to conclude the directionality of causation—and to separate whether the males or the females drive these relationships—would require experimental manipulation of mate fidelity, site fidelity, or both.

Together, our results support theoretical arguments that divorce is an adaptive strategy by which individuals improve their reproductive success (Black, 1996; Dubois & Cézilly, 2002; McNamara & Forslund, 1996). Divorced birds reached higher number of breeding attempts and higher breeding success than individuals that retained their mates, at least within years. We suggest that in snowy plovers, divorce is result from their effort to maximize reproductive output during a given time period. The birds' urge to re‐mate as many times as possible within a breeding season and produce the highest possible number of chicks could be traded off by lowered survival of their abandoned broods although, given the precociality of the young, this cost may not be prohibitive (Székely & Williams, 1995). We suggest that the urge for a fast reproduction in snowy plovers is an adaptive response to life histories (i.e., short life span) and breeding parameters (i.e., short breeding period and breeding success). Additionally, time constraint in breeding confounded with the bias in population demography (i.e., male‐biased adult sex ratio) propels both sexes adopt different mating strategies, resulting in different spatial dispersal patterns. Therefore, mate choice and breeding dispersal are important components of their breeding strategy. We encourage further investigations of breeding strategy including mate fidelity between different polygamous shorebird populations and to understand the generality of our findings across the various natural populations with the intention of informing conservation decisions.

## CONFLICT OF INTEREST

We have no conflict of interest to declare.

## AUTHORS' CONTRIBUTIONS

N. H., P. H., and T. S. conceived the project; K. K., M. A. S.‐M., and M. C.‐L. provided the data; N. H. and K. K. carried out the statistical analyses. All authors contributed critically to the drafts and gave final approval for publication.

## ETHICS STATEMENT

All aspects of the fieldwork were authorized by the national authorities of Mexico (Secretaría de Medio Ambiente y Recursos Naturales, SEMARNAT; SGPA/DGVS/01717/10, SGPA/DGVS/01367/11). Birds were ringed and handled by trained people aiming to cause as little disturbance to birds as possible.

## Data Availability

Data are available from the Dryad Digital Repository, https://doi.org/10.5061/dryad.3185h66
